# miR-9-5p as a Regulator of the Androgen Receptor Pathway in Breast Cancer Cell Lines

**DOI:** 10.3389/fcell.2020.579160

**Published:** 2020-11-12

**Authors:** Erika Bandini, Francesca Fanini, Ivan Vannini, Tania Rossi, Meropi Plousiou, Maria Maddalena Tumedei, Francesco Limarzi, Roberta Maltoni, Francesco Fabbri, Silvana Hrelia, William C. S. Cho, Muller Fabbri

**Affiliations:** ^1^Biosciences Laboratory, Istituto Scientifico Romagnolo per lo Studio e la Cura dei Tumori (IRST) IRCCS, Meldola, Italy; ^2^Istituto Scientifico Romagnolo per lo Studio e la Cura dei Tumori (IRST) IRCCS, Meldola, Italy; ^3^Department for Life Quality Studies, Alma Mater Studiorum - University of Bologna, Rimini, Italy; ^4^Department of Clinical Oncology, Queen Elizabeth Hospital, Kowloon, Hong Kong; ^5^Cancer Biology Program, University of Hawai’i Cancer Center, Hawaii, HI, United States

**Keywords:** microRNAs, miR-9-5p, androgen receptor, breast, cancer

## Abstract

Breast cancer (BC) is the most diagnosed carcinoma and the leading cause of cancer death in female over 100 countries. Thanks to the advance in therapeutic strategies, patients’ survival has improved. However, the lack of response to treatments and drug resistance are still a main concern, demanding for new therapeutic approaches, in particular for the advanced stages of the disease. Androgen receptor (AR) is gaining increasing interest as a fourth targetable receptor in BC, however, its regulation in BC cells is still poorly understood. MicroRNAs (miRNAs) regulate gene expression post-transcriptionally. Here, we identified miR-9-5p as an inhibitor of AR expression, we validated the inverse correlation between miR-9-5p and AR in primary BC samples and we further identified a feedback loop in which androgen agonists of AR up-regulate miR-9-5p. We also provided evidence that miR-9-5p elicits anti-proliferative effects in BC cell lines regardless of their estrogen receptor status. Finally, we showed that miR-9-5p can revert AR-downstream signaling even in presence of AR-agonists, highlighting the role of this miR in the hormonal response of BC. In conclusion, this study supports the role of miR-9-5p as an anti-proliferative miR in BC and as a central modulator of AR-signaling response to circulating androgens in BC.

## Introduction

Despite progresses in its global management approaches, breast cancer (BC) still remains the most diagnosed carcinoma and the leading cause of cancer death in women over 100 countries (Bray et al., 2018). In 2020 it was estimated an incidence of 276,480 cases and 42,170 deaths in women due to BC in the United States (Siegel et al., 2020). Although several therapeutic approaches have provided remarkable improvements in survival, drug resistance and the lack of response to treatments are still a main issue, suggesting a great need to exploit novel therapeutic strategies in particular in the advanced stages (Zhao et al., 2019). Androgen receptor (AR) is a member of the steroid nuclear receptors family that mediates the biological effects of androgens. It is considered an emerging target in the treatment of patients characterized by the lack of expression of hormone receptors (the so called “Triple-Negative Breast Cancer” or TNBC), and in other BC subtypes, despite its role is still controversial (Takagi et al., 2018; Vidula et al., 2019). In recent years, several molecules have been investigated as crucial regulators of transcription, and translation of genes involved in carcinogenesis, and microRNAs (miRNAs) are among the most studied. They belong to a large family of small non-coding RNAs and deserve great attention since they can modulate the expression of tumor suppressor genes and oncogenes, affecting the signal pathways in cancer cells. However, little is known about miRNA regulation of hormonal signaling, especially androgen signaling, in BC, and how this affects hormone-therapy. Several studies have been performed in order to identify a miRNA signature that could predict their potential role as prognostic or predictive biomarkers for patient management. In particular, miR-9-5p dysregulation has been reported in many cancer types, but its role as a tumor suppressor gene or as an oncomiR remains unclear, especially in BC. Some studies reported miR-9-5p as a negative prognostic marker related to more aggressive clinical pathological features and as involved in epithelial-mesenchymal transition (EMT) (Almeida et al., 2010; Gwak et al., 2014; van Schooneveld et al., 2015; Bastos et al., 2016; Jang et al., 2017; Cheng et al., 2018). AR has emerged as a fourth receptor in BC and as a promising target, especially for those patients who do not respond to current hormone receptor-targeted therapies. In this study, we investigated the role of miR-9-5p in the AR pathway. We showed that AR is a target of miR-9-5p and we identified a novel miR-9-5p/AR feedback loop with important implications for the response of BC to anti-androgen therapy.

## Materials and Methods

### Cell Cultures

The human breast carcinoma cell lines MDA-MB-453 and MCF-7 were purchased from the American Type Culture Collection (ATCC; Manassas, Virginia, United States), and the cell line T-47D was purchased from Zooprophylactic Institute of Genova (Italy). MDA-MB-453 were maintained in Leibovitz’s L-15 medium (ATCC 30-2008, United States) supplemented with fetal bovine serum (FBS) (Gibco, Thermo Fisher Scientific, United States) to a final concentration of 10%, according to the manufacturer’s information sheet. MCF-7 was maintained in Eagle’s Minimum Essential Medium (EMEM) medium (ATCC 30-2003, United States) supplemented with FBS to a final concentration of 10%. T-47D were maintained in Dulbecco’s Modified Eagle’s Medium (DMEM) High Glucose (Euroclone, Italy), and FBS to a final concentration of 10%. One percent of Penicillin-streptomycin (PAA, Carlo Erba Reagents, Italy), and 0.002% of MycoZap Prophylactic (Lonza Group Ltd., Switzerland) were added to all mediums. The cultures were maintained in a Heraeus incubator, in atmosphere composed of 95% air, and 5% CO_2_, except for MDA-MB-453 that required a free gas exchange with atmospheric air. Every 4 days we proceeded to sub-cultivation of cell lines by using Trypsin-EDTA (Life Technologies, United States). Cell lines were tested every 2 months with MycoAlert^TM^ Mycoplasma Detection Kit (Lonza Group Ltd., Switzerland) to check a possible contamination by mycoplasma. In experiments where hormonal treatments were performed, medium phenol red free added of FBS charcoal stripped was used (Gibco, Thermo Fisher Scientific, United States).

### Patient Sample

Paired FFPE tumor and adjacent non-tumor tissue from 11 BC patients (5 Triple Negative Breast Cancers TNBCs, and 6 Luminal A) were obtained at the Istituto Scientifico Romagnolo per lo Studio e la Cura dei Tumori IRST, S.r.l, IRCCS, in Meldola (FC), Italy. Histological and clinical characteristics are listed in Supplementary Table S1. Written informed consent was obtained from all patients before sample analyses. The study was approved by the Ethical Committee of our institute and conducted in accordance with the Declaration of Helsinki. Written informed consent was obtained from all patients before the first sampling.

### Drug Treatments

Dihydrotestosterone (DHT) was purchased from Sigma-Aldrich (Merck, Germany) in powder formulation and a 10 μM working solution was obtained by resuspending it in dimethyl sulfoxide (DMSO); DHT was then used at 10 nM and DMSO never exceeded 0.1%. Dehydroepiandrosterone (DHEA) was provided by the Pharmacy of our Institute at a concentration of 7.6 mM dissolved in sterile water, and 50 μM solution was used to perform experiments.

### Pre-miRNAs Transfection

Pre-miRNAs (pre-miRNA miR precursors, Ambion, Life Technologies, United States), and the corresponding negative control, SCR (Pre-miR miRNA precursor scrambled negative control #1, Ambion, Life Technologies, United States) were used to transfect BC cell lines at a final concentration of 100 nM through TransIT-X2^®^ Dynamic Delivery System (Mirus Bio LLC, United States), in accordance with the instructions provided by the manufacturer. Opti-Mem Medium (Gibco, Thermo Fisher Scientific, United States) was used for transfection complex. Depending on the type of final analysis, transfections were stopped and pellets collected at 24–48–72 h. MiRNA transfection efficiencies were evaluated by Real-time quantitative PCR (qRT-PCR).

### AR Silencing

SMARTpool siGENOME AR siRNA (Dharmacon), and the corresponding negative control, siGENOME Non-Targeting siRNA #1 (Dharmacon), were transfected into BC cell lines at a final concentration of 50 nM, through TransIT-X2^®^ Dynamic Delivery System (Mirus Bio LLC, United States), in accordance with the instructions provided by the manufacturer. Opti-Mem Medium (Gibco, Thermo Fisher Scientific, United States) was used for transfection complex. Transfections were stopped and pellets collected at 48 h. AR transfection efficiencies and miR-9-5p expression were evaluated by qRT-PCR.

### Extraction of RNA and Proteins

RNA was isolated from BC cells with mirVana^TM^ miRNA Isolation Kit (Invitrogen, Thermo Fisher Scientific, United States), following the manufacturer’s instruction. RNA isolation from 2-to-4 10 μm thick slices FFPE was performed by using RecoverAll Total Nucleic Acid Isolation Kit for FFPE (Invitrogen, Thermo Fisher Scientific, United States) following the protocol provided by the manufacturer. The quantification of extracted RNA was carried out using NanoDrop ND-1,000 (Thermo Fisher Scientific, United States). Total proteins were extracted, keeping samples on ice, with 1X RIPA lysis buffer (Santa Cruz Biotechnology, United States) with the addition of 10 μl of PMSF, 10 μl of sodium orthovanadate and 15 μl of protease inhibitors per ml of 1X RIPA lysis buffer, as recommended by the datasheet. The lysates were centrifuged at 4°C for 30 min. Then, supernatant was transferred to another tube. Proteins were quantified through BCA Protein Assay (Pierce, Thermo Fisher Scientific, United States), and using a Multiscan EX microplate reader (Thermo Fisher Scientific, United States), at a wavelength of 490 nm.

### Protein Expression Analysis

Western blotting was used to evaluate the expression of AR. Proteins (50 μg) were denatured and separated by electrophoresis using a gel Criterion TGX Stain Free Gel Precast 4–20% (Bio-Rad Laboratories, CA, United States), and Laemmli Sample Buffer (Bio-Rad Laboratories, CA, United States) with 5% of β-mercaptoethanol (Carlo Erba Reagents, Italy), in 1:1 ratio with the sample. Electrophoretic run was performed at a constant voltage of 180V in a TRIS/Glycine/SDS 1X buffer (Bio-Rad Laboratories, CA, United States). Trans-Blot Transfer Turbo midi-format 0.2 μm PVDF membrane (Bio-Rad Laboratories, CA, United States) using the Trans Blot Turbo System (Bio-Rad Laboratories, CA, United States). The membrane was subsequently incubated for at least 2 h at room temperature in a solution of Tween 20 (Bio-Rad) at 0.1% and 1X Dulbecco’s phosphate buffered saline (DPBS) (Invitrogen, Thermo Fisher Scientific, United States) supplemented with 5% nonfat dry milk (Bio-Rad Laboratories, CA, United states) in order to facilitate the saturation of non-specific binding sites. Primary antibodies and dilutions used are the following: anti-Androgen Receptor (1:1,000; ab133273, Abcam, United States), anti-Vinculin clone FB11 (1:1,000; Biohit, United Kingdom), anti-β-actin Antibody HRP (1:50,000; ab49900, Abcam, United States). Secondary antibodies HRP-conjugated goat anti-mouse and anti-rabbit (1:5,000; Santa Cruz, United States) and Precision Plus Protein Western C Strep Tactin-HRP Conjugate (1:10,000; Bio-Rad Laboratories, CA, United States) were used. The secondary HRP-conjugated antibodies were used for 1 h at room temperature. The membranes, after washes with T-PBS, were developed using Clarity Western ECL reagent (Bio-Rad Laboratories), and images acquired through Chemidoc (Bio-Rad Laboratories), and analyzed using ImageJ Software (Wayne Rasband, NIH, United States).

### miRNA and mRNA Expression Analysis

MiRNAs expression analysis was performed using the TaqMan miRNA Assays (Applied Biosystems, Thermo Fisher Scientific, United States). Briefly, the molecule of complementary DNA (cDNA) was synthesized using 10 ng of RNA as template, specific primer and TaqMan MicroRNA Reverse Transcription Kit (Applied Biosystems, Thermo Fisher Scientific, United States). mRNAs expression was evaluated with the use of the TaqMan Gene Expression Assays (Applied Biosystems, Lifetechnologies, United States). The cDNA was synthesized using 80 ng of RNA as a template and the TaqMan RT PCR Kit (Applied Biosystems, Thermo Fisher Scientific, United States). qRT-PCR was performed with Applied Biosystems 7500 Real-Time PCR System (Thermo Fisher Scientific, United States) using cDNA, TaqMan probes and TaqMan Universal PCR Master Mix (Applied Biosystems, Thermo Fisher Scientific, United States). To determine the basal expression of miR-9-5p in BC cell lines, a reference RNA composed of total RNA from nine human tissues or cell lines was used (Total RNA Breast Human, #750500, Agilent Technologies, United States). Experiments were conducted in triplicate and normalized to RNU48, RNU44 and GAPDH, used as endogenous controls. Relative expression levels were calculated using the method of comparative Ct (2^–Δ^ ^Δ^ ^*Ct*^ method).

### Immunohistochemistry

During surgery some tumor samples were obtained and fixed in neutral buffered formalin, then embedded in paraffin. For each patient, 5 μm thickness sections were mounted on positive-charged slides (Bio Optica, Milan, Italy). AR expression was performed using the Ventana Benchmark ULTRA staining system (Ventana Medical Systems, Tucson, AZ, United States) with the Optiview DAB Detection Kit (Ventana Medical Systems). AR (SP107 Cell Marque, Ventana Medical Systems) antibody pre-diluted by the supplier was used. The slides were incubated for 16 min and automatically counterstained with hematoxylin II (Ventana Medical Systems). AR positivity was detected and semiquantitatively quantified as the percentage ratio between immunopositive tumor cells and the total number of tumor cells. All samples were evaluated by two independent observers. Any disagreement (>10% of positive cells for the different markers) was resolved by consensus after joint review using a multihead microscope. Biomarker determination was performed according to European Quality Assurance guidelines

### CellTiter-Glo Luminescent Cell Viability Assay

CellTiter-Glo Luminescent Cell Viability Assay (Promega, United States) was used to determine the number of viable cells after hormone treatments and transfections. It is based on the quantification of present ATP that indicates the existence of metabolically active cells. The procedure, according to the instructions provided by the manufacturer, requires the adding of a single reagent (CellTiter-Glo Reagent) directly to cultured cells. This system produces a cell lysis and the generation of a luminescent signal, captured through a GloMax luminometer (Promega, United States), proportional to the ATP content, which, in turn, is directly proportional to the number of viable cells.

### Flow Cytometry

All samples were analyzed by using a FACSCanto^TM^ cytofluorimeter (BD Biosciences). Data acquisition (10,000 events were collected for each sample) was performed by using the FACS Diva^TM^ software (BD Biosciences), as recommended by the manufacturer. After cells were seeded in 6-well plates and treated with DHT 10 nM or DHEA 50 μM, or after miR-9-5p transfection they underwent different protocols.

Bromodeoxyuridine assay was used to determine the percentage of S-phase cells. Cells were incubated with a 60 μM Bromodeoxyuridine (BrdU) solution in 1 ml of medium and then centrifuged and fixed with 70% cold ethanol. The day after cells were washed in PBS 1X and incubated with the following reagents in this order: HCL 2M (Carlo Erba Reagents, Italy), sodium tetraborate (Sigma-Aldrich, Merck, Germany), and Tween 20 0.5% (Bio-Rad Laboratories, CA, United States) + BSA 1% (Thermo Fisher Scientific, United States). Finally, cells were incubated with a 1:50 dilution of anti-BrdU antibody (Thermo Fisher Scientific, United States) for 1 h, followed by 1 h of a FITC-conjugated secondary antibody (Dako, Agilent Technologies, United States) diluted 1:250. After antibody incubation, cells were washed with PBS 1X, stained with propidium iodide solution and incubated overnight at 4°C. Samples were processed and analyzed the day after.

For cell cycle evaluation, cells were harvested after each treatment timepoint, fixed in 70% ethanol, and stained in a solution containing 10μg ml/1 of propidium iodide (Sigma-Aldrich), 10,000 U ml/1 of RNase (Sigma-Aldrich), and 0.01% of NP40 (Sigma-Aldrich). After 30–60 min, the samples were analyzed. Data were elaborated using the ModFit LT^TM^ software v.4.1.7 (Verity Software House), and expressed as fractions of cells in the different cell-cycle phases.

### Cignal Reporter Assay

Cignal Reporter Assay Kit (Qiagen, United States) was used to assess the activation of AR transduction pathway, by measuring the activities of downstream AR transcription factors (both increases and decreases). One microgram of AR-responsive reporter (a mixture of inducible transcription factor responsive construct and constitutively expressing Renilla luciferase construct) and negative control (a mixture of non-inducible reporter construct and constitutively expressing Renilla luciferase construct), along with pre-miR-9-5p and the corresponding negative control SCR (100 nM), were diluted in Opti-Mem Medium (Gibco, Thermo Fisher Scientific, United States). The diluted nucleic acids were mixed with TransIT-X2 Mirus (Temaricerca, Italy). Following 24 h transfection, cells were treated with DHT 10 nM for additional 24 h, so that 48 h post-transfection cells were harvested into cell lysis buffer (Promega, United States). Luciferase activity was measured using the Dual-Luciferase Assay System (Promega, United States). The change in the activity of AR signaling pathway was determined by comparing the normalized luciferase activities of the reporter in treated *versus* untreated transfectants (SCR vs. miR-9-5p and SCR + DHT vs. miR-9-5p + DHT). The activity ratio Firefly:Renilla was calculated from experimental and control transfections. Then, ratios from AR responsive reporter transfections were divided by ratios from negative controls to obtain relative luciferase units (RLU).

### Data Analysis

Data obtained from qRT-PCR were analyzed by Life Technologies TM 7500 Software v2.0.6 for 7500 Fast Real Time PCR System (Life Technologies, Thermo Fisher Scientific, United States). Relative expression levels were calculated using the method of comparative Ct (2^–Δ^ ^Δ^ ^*Ct*^). The images of the Western Blot, acquired through Chemidoc, were analyzed using ImageJ Software (Wayne Rasband, NIH, United States). Viability experiments were conducted in 96 wells plate, using 8 wells for every sample. Statistical analyses were performed using GraphPad Prism 8 (version 8.4.2) statistical software (GraphPad Software, United States). Statistical significance was indicated as follows: ^∗^*p* < 0.05; ^∗∗^*p* < 0.01; ^∗∗∗^*p* < 0.001.

## Results

### MicroRNA-9-5p Is Downregulated in BC Cell Lines

**FIGURE 1:**
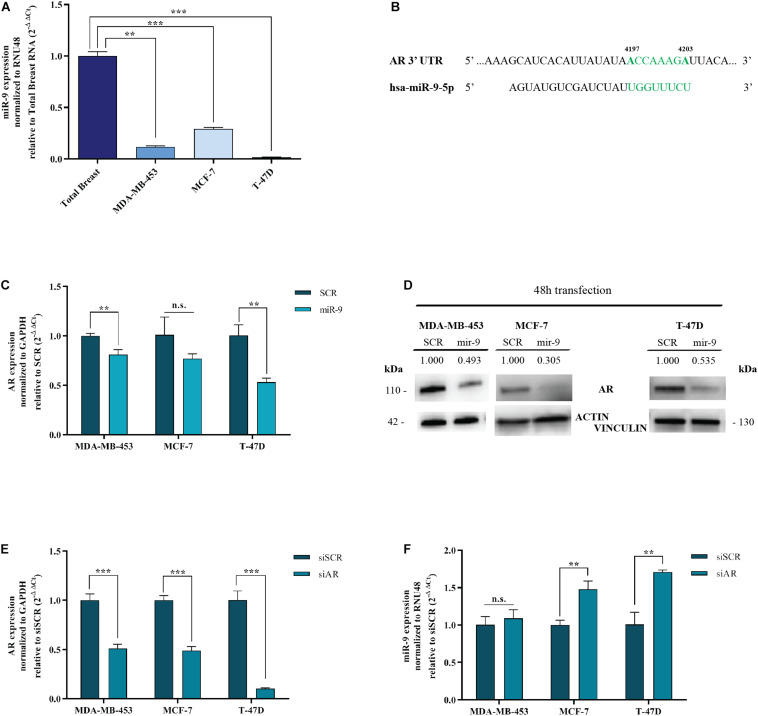
MiR-9-5p is downregulated in BC cell lines and down-regulates AR. **(A)** MiR-9-5p (mir-9) expression was measured by qRT-PCR in three BC cell lines and compared to Total Breast RNA. **(B)** Predicted miR-9-5p target sites in the AR 3′UTR mRNA, as shown by the Software TargetScan Human Release v6.2. in green, the seed region of miR-9::AR mRNA predicted interaction. **(C)** mRNA expression level of AR in MDA-MB-453, MCF-7, and T-47D cell lines, 48 h after transfection with pre-miR-9-5p (miR-9), or scrambled (SCR). **(D)** Protein expression level of AR in MDA-MB-453, MCF-7 and T-47D cell lines, 48 h after transfection with pre-miR-9-5p (miR-9), or scrambled (SCR). **(E)** AR levels after transfection of MDA-MB-453, MCF-7, and T-47D cell lines with AR siRNA (siAR) compared to scrambled (siSCR). **(F)** MiR-9-5p (miR-9) expression in MDA-MB-453, MCF-7, and T-47D cell lines after 48 h transfection with AR siRNA (siAR) or scrambled (siSCR). **p < 0.01, ***p < 0.001, n.s., not significant. Multiple *t*-test, corrected for multiple comparison using the Holm-Sidak method in **(A–D)**.

MDA-MB-453 are molecular apocrine BC cell lines (AR^+^, ER^–^) (Robinson et al., 2011). MCF-7 and T-47D are luminal A BC cell lines (AR^+^, ER^+^) (Dai et al., 2017). We performed qRT-PCR for miR-9-5p to assess the endogenous expression of miR-9-5p in these 3 different BC cells lines, compared to human Total Normal Breast RNA (Agilent Technologies). All cell lines show a low endogenous expression of miR-9-5p when compared to Total Normal Breast RNA (Figure 1A).

### MicroRNA-9-5p Silences AR

AR is an emerging target for hormone therapy in BC. However, the factors regulating its expression are not understood. Intriguingly, miR-9-5p is predicted to target AR by TargetScan Software v7.2 (Agarwal et al., 2015) and miRWalk database (Medical Faculty Mannheim of the University of Heidelberg) (Figure 1B). To assess whether miR-9-5p silences AR, MDA-MB-453, MCF-7 and T-47D cells were transfected with miR-9-5p or a scrambled oligonucleotide as a control (SCR) and the expression of AR was measured both at the mRNA and at the protein level by qRT-PCR and immunoblotting, respectively. We observed a significant down-regulation of AR expression in all three tested BC cell lines both at the mRNA (Figure 1C) and at the protein level (Figure 1D).

### AR Silencing Upregulates miR-9-5p in ER+ Cell Lines

Intriguingly, when we silenced AR endogenous expression with a pool of siRNAs (a mixture of 4 siRNA provided as a single reagent) (Figure 1E), upregulation of miR-9-5p was observed (compared to a siRNA negative control) but only in ER+ BC cell lines MCF-7 and T-47D, with no effects on the ER- cell line MDA-MB-453 (Figure 1F). Overall, these data indicate that while miR-9-5p downregulates AR expression regardless of the ER status of BC cell lines, AR silencing up-regulates miR-9 only in ER^+^ cell lines.

### MicroRNA-9-5p Reduces BC Cell Growth

**FIGURE 2:**
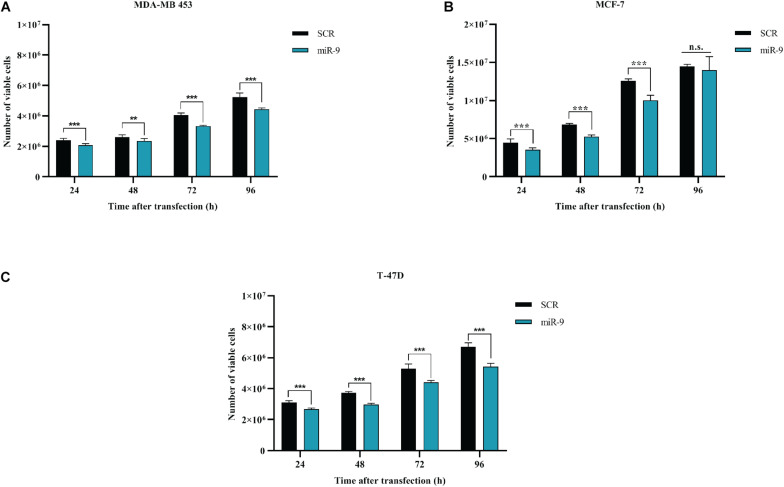
Effects of miR-9-5p (mir-9) on proliferation of BC cell lines **(A)** MDA-MB-453 **(B)** MCF-7 **(C)** T-47D. **p < 0.01, ***p < 0.001, n.s., not significant. Multiple *t*-test, corrected for multiple comparison using the Holm-Sidak method in **(A–C)**.

**FIGURE 3:**
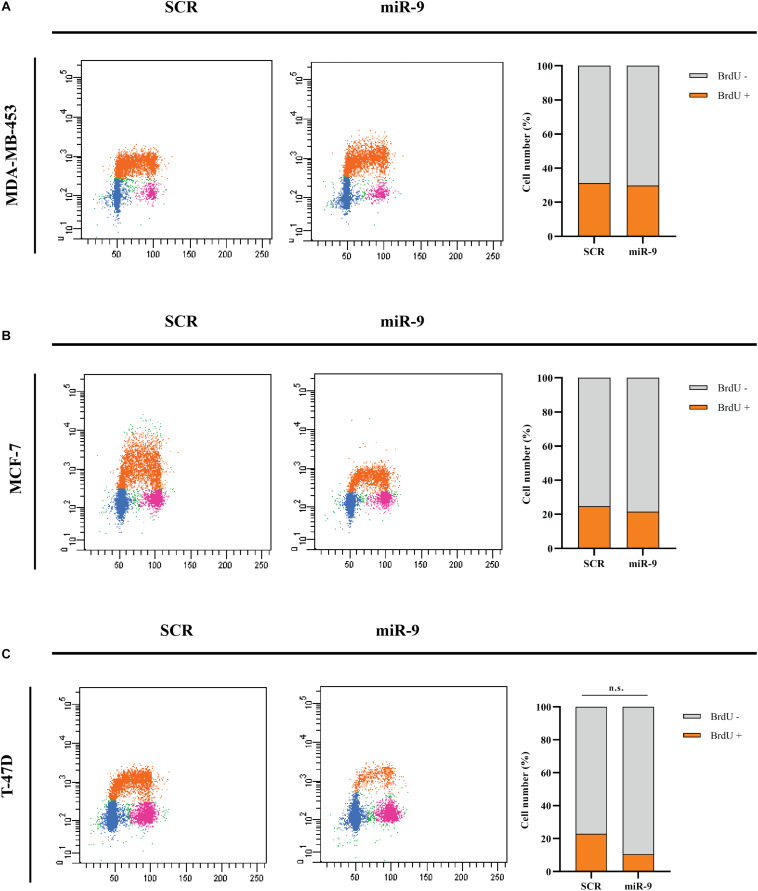
Cytofluorimetric evaluation of the effects of miR-9-5p (miR-9) on DNA replication at 72 h post-transfection. Cytofluorimetric dot plots of proliferating MDA-MB-453 **(A)**, MCF-7 **(B)**, and T-47D **(C)** BC cell lines, following BrdU incorporation, and anti-BrdU antibody incubation. The percentage of cells in S phase, which results in BrdU positive cells (BrdU+) was presented in the histograms on the right.

BC cells were transfected with the pre-miR-9-5p mimic, or a scrambled miRNA as a control (SCR), and *in vitro* cell growth at 24–48–72–96 h post-transfection was evaluated. MiR-9-5p increased expression significantly reduced the proliferation of all three BC cells at all tested times (Figures 2A–C). Overexpression of miR-9-5p was verified by qRT-PCR (Supplementary Figures S1A–C). In order to study the effects of mir-9-5p on cell proliferation, we further quantified replicating DNA by performing a BrdU assay. While only little difference was observed in MDA-MB-453 and MCF-7 cell lines at 72 h (Figures 3A,B), we observed a notable reduction of DNA replication in T-47D cell line transfected with pre-miR-9-5p compared to scrambled (25 vs. 6.2%) at 72 h (Figure 3C) which persisted even at 96 h (Supplementary Figure S2), suggesting that cells did not enter S phase. These results were confirmed also by cell cycle analysis using propidium iodide, which showed a reduction of T-47D cells in S-phase and a block in G1-phase when miR-9-5p was overexpressed (Supplementary Figures S3–S5).

### AR Silencing Reduces Proliferation of ER+ Cells Lines

**FIGURE 4:**
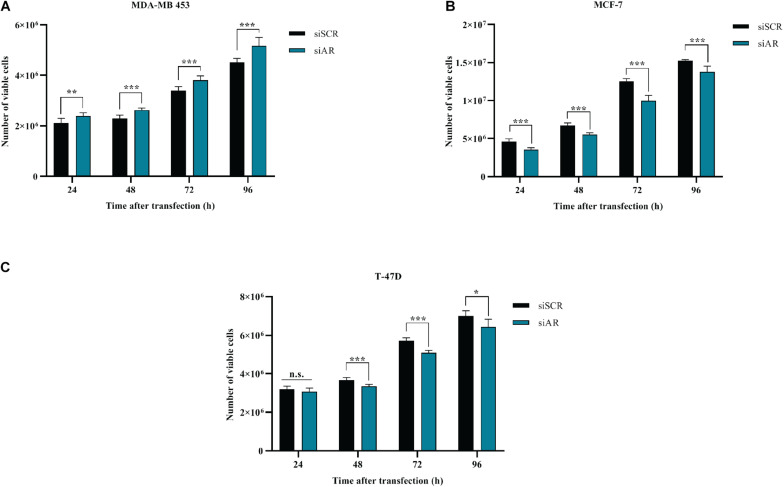
Effects of AR silencing (siAR) on proliferation of BC cell lines **(A)** MDA-MB-453 **(B)** MCF-7 **(C)** T-47D. *p < 0.05, **p < 0.01, ***p < 0.001, n.s., not significant. Multiple *t*-test, corrected for multiple comparison using the Holm-Sidak method in **(A–C)**.

BC cells were transfected with a pool of AR siRNAs (a mixture of 4 siRNA provided as a single reagent) (Figure 1E) and *in vitro* cell growth at 24–48–72–96 h post-transfection was assessed. AR silencing did not reduce the proliferation of ER- MDA-MB-453 (Figure 4A), but significantly reduced the proliferation of the two ER+ cell lines MCF-7 and T-47D (Figures 4B,C). These data are consistent with the inhibitory effect of miR-9-5p on BC proliferation and with the fact that silencing of AR expression up-regulates miR-9-5p only in ER+ cells and not in the ER- MDA-MB-453 BC cells.

### DHT and DHEA Reduce Growth of BC Cell Lines

**FIGURE 5:**
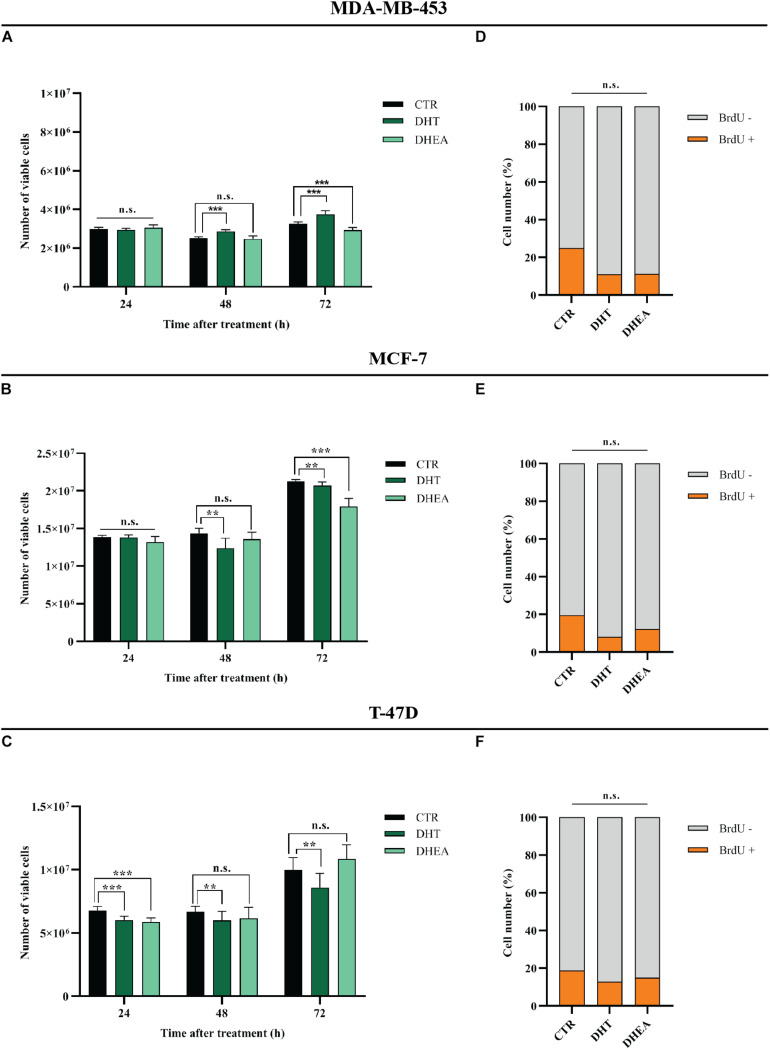
Effect of DHT and DHEA on proliferation of BC cell lines at the indicated time points **(A–C)** and on DNA replication at 48 h post-transfection **(D–F)**. **p < 0.01, ***p < 0.001, n.s., not significant. Multiple *t*-test, corrected for multiple comparison using the Holm-Sidak method in **(A–F)**.

MDA-MB-453, MCF-7, and T-47D cell lines express AR (Figure 1D). In order to assess the effects of circulating AR-agonists on BC cell proliferation, we treated BC cells with DHT and DHEA, respectively. DHT showed a cell growth promoting effect only in MDA-MB-453 at 48 and 72 h (Figure 5A), while it displayed a significant inhibitory effect in MCF-7 at 48 and 72 h (Figure 5B) and in T-47D at all tested time-points (Figure 5C). Nevertheless, BrdU assay indicated a reduction trend in percentage of cells in S phase for all three cell lines tested, although the statistical analysis did not yield any significant differences between treated and untreated group (Figures 5D–F). DHEA showed inhibition of cell growth in MDA-MB-453 and MCF-7 cell lines at 72 h (Figures 5A,B) and in T-47D only at 24 h after treatment (Figure 5C). Similarly to DHT-treated cells, also for DHEA-treated cells the BrdU assay showed a decrease of cell number in S phase for all three tested cell lines although the statistical analysis did not yield any significant differences between the two groups (Figures 5D–F).

### DHT and DHEA Up-Regulate miR-9-5p

**FIGURE 6:**
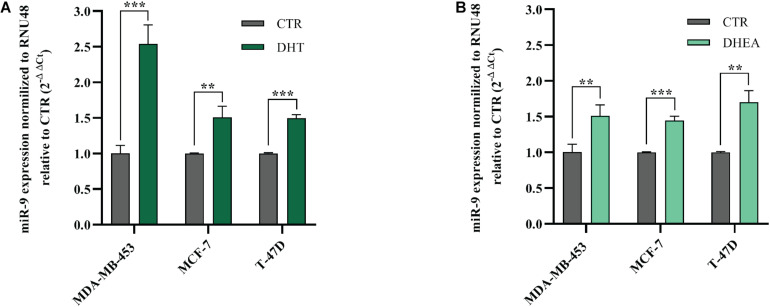
Effects of DHT and DHEA on miR-9-5p (miR-9) expression at 24 h post-transfection **(A,B)**. **p < 0.01, ***p < 0.001. Multiple *t*-test, corrected for multiple comparison using the Holm-Sidak method in **(A,B)**.

Next, we decided to assess whether AR agonists DHT and DHEA were able to modulate miR-9-5p expression, which is endogenously low in the three BC cell lines included in this study, compared to normal breast cells (Figure 1A). We treated MDA-MB-453, MCF-7, and T-47D cells with DHT or DHEA, and we observed that miR-9-5p was significantly upregulated in all cell lines both by DHT (Figure 6A) and DHEA (Figure 6B). Since miR-9-5p down-regulates AR protein expression (Figure 1D), we conclude that a feedback loop occurs between miR-9-5p and AR, in which AR agonists can induce miR-9-5p expression which, in turn, inhibits AR protein levels in all three BC cell lines and regardless of their ER status.

### MicroRNA-9 Inhibits AR Transcriptional Activity

**FIGURE 7:**
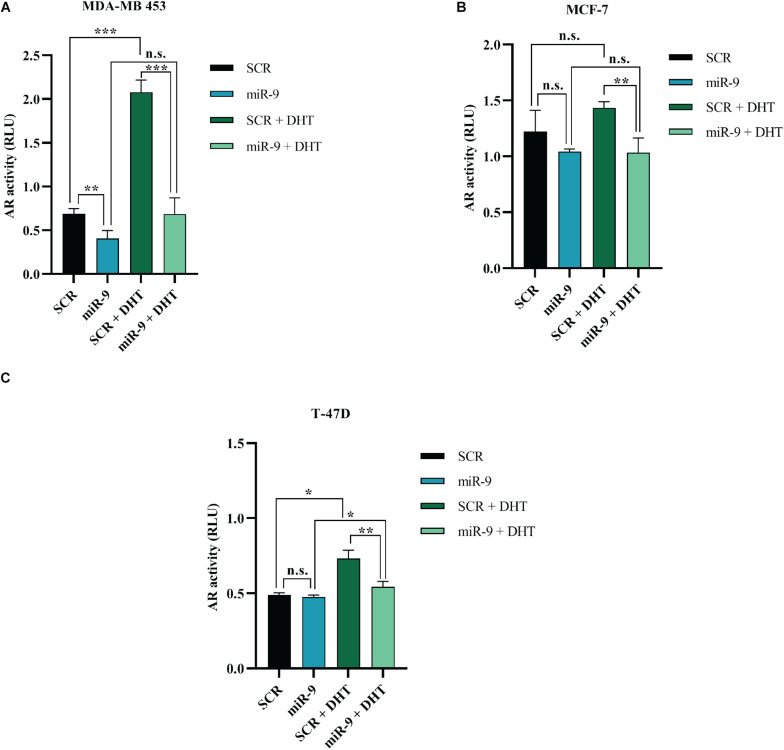
Effects of miR-9-5p (mir-9) on AR signaling. AR transcriptional activity as measured with the AR Cignal reporter assay at 48 h in MDA-MB-453 **(A)**, MCF7 **(B)**, and T47D **(C)** cells. The modulation of AR transcription factor was determined by comparing the normalized luciferase activities of the reporter in treated vs. untreated transfectants (SCR vs. miR-9, SCR vs. SCR + DHT, miR-9 vs. miR-9+DHT, and SCR+DHT vs. miR-9+DHT), in all the BC cell lines MDA-MB-453 **(A)**, MCF-7 **(B)**, and T-47D **(C)**. Firefly: Renilla activity ratios were calculated from experimental and control transfections. Then, ratios from AR responsive reporter transfections were divided by ratios from negative controls to obtain relative luciferase units (RLU). *p < 0.05, ** *p* < 0.01, ***p < 0.001, n.s., not significant. Multiple *t*-test, corrected for multiple comparison using the Holm-Sidak method in **(A–C)**.

It has been shown that miR-9-5p silences AR in BC cell lines. In order to assess whether this reflects in an effect of miR-9-5p on AR-mediated downstream signaling, we performed the AR Cignal Reporter Assay. We observed that while miR-9-5p over-expression had minimal effects on the AR signaling (as measured by the luciferase activity of the Cignal reporter plasmid) and in absence of AR agonists (DHT and DHEA), miR-9-5p over-expression was able to reverse the activation of AR signaling even in presence of AR agonists (Figures 7A–C). These findings suggest that miR-9-5p has a leading effect in shaping AR-mediated downstream pathway and that the effects of miR-9-5p on the AR downstream signaling mostly occur in presence of the AR agonists (Figures 7A–C).

### MicroRNA-9 and AR Expression Show an Inverse Correlation in BC Patients

**FIGURE 8:**
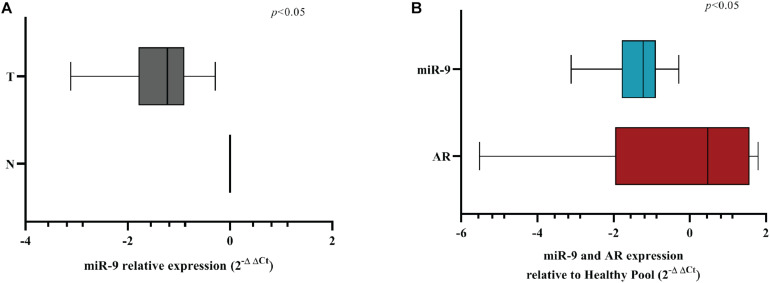
MiR-9-5p is downregulated in BC FFPE samples. **(A)** Box-plot representation of the miR-9-5p (miR-9) qRT-PCR expression in adjacent normal (N) vs. tumoral (T) samples for all FFPE samples examined (*n* = 11). **(B)** Box-plot representation of the miR-9-5p (miR-9) and AR qRT-PCR expression in all FFPE samples examined (*n* = 11). The expression of AR and miR-9 have been normalized to GAPDH and RNU44, respectively. A non-parametric unpaired *t*-test (Kolmogorov-Smirnov test) method in (**A**,**B)**. Box-plots have been presented on a ln-scale.

In order to evaluate if miR-9-5p was downregulated in BC patients, we performed a qRT-PCR analysis in 11 primary BC samples. Results showed that miR-9-5p was significantly downregulated in tumor tissues (T) compared to healthy adjacent ones (N) in all patients (Figure 8A), and an inverse correlation between miR-9-5p and AR expression was observed in tumor tissues (Figure 8B) validating in patient samples our findings that miR-9-5p is a regulator of AR expression in BC. Expression of AR in FFPE samples was confirmed by immunohistochemistry (IHC) (Supplementary Figures S6A,B).

## Discussion

In the last few years, AR has emerged as a new potential target for the treatment of BC patients. Indeed, circulating androgens are present at physiological conditions in women, and their levels vary depending on hormonal needs and in relation to pre- or post-menopausal state (Giovannelli et al., 2018). Approximately 50–80% of BCs are positive for AR, but the prognostic and predictive value of its expression in BC is still controversial (Yang et al., 2020). MiRNAs are increasingly implicated in regulating tumorigenesis of BC, and among them miR-9-5p is attracting great attention. Its role is currently still debated since it has shown functions both as an oncomir and as a tumor suppressor gene (Gravgaard et al., 2012; Selcuklu et al., 2012; Orangi and Motovali-Bashi, 2019; Sporn et al., 2019). The current evidence points toward a BC stage-dependent role of miR-9-5p: as a tumor suppressor gene in the early stage of BC, and as a promoter of the metastatic progression, essentially playing as an oncogene, at later stages (Li et al., 2020). For instance, Gravgaard et al. (2012) observed that miR-9-5p may be involved in the metastatic process, with a higher expression in distant metastases compared to the corresponding primary tumor. Low miR-9-5p expression was also found to be associated with a better prognosis, smaller tumors, earlier stage and ER+ cancers (Sporn et al., 2019). Controversial studies attributed downregulated miR-9-5p to anti-proliferative and pro-apoptotic functions in BC cells compared to healthy ones (Selcuklu et al., 2012; Orangi and Motovali-Bashi, 2019). Others described an unclear role of miR-9-5p in tumor tissues and BC cells, suggesting that its function may depend on different subtypes of BC or progression level (Krell et al., 2012; Hasanzadeh et al., 2016; Shi et al., 2017). Curiously, a group reported different expression levels of miR-9-5p in tissues compared to serum of BC patients (Tavakolian et al., 2019). Previous findings revealed that miR-9-5p expression can be influenced by steroid hormone receptors, and that it is implicated in a regulatory mechanism signaling in BC, besides being involved in affecting the biology of the tumor microenvironment (Zhuang et al., 2012; Pillai et al., 2014; Baroni et al., 2016; Barbano et al., 2017). Interestingly, Baldassari et al. (2018) demonstrated that a combined treatment with miRNAs, including miR-9-5p, enhances the activity of specific anti-BC drugs *in vitro*, even on the most aggressive HER2+ and TNBC subtype. However, the functional implications of miR-9-5p in AR signaling have not yet been deeply explored in BC. Today, among the main therapies for advanced BC in Tamoxifen-resistant BCs and TNBCs, are Bicalutamide and Enzalutamide, first and second generation AR-directed antagonists (Salvi et al., 2019). One of the first phase II trials involving Bicalutamide revealed evidence of androgen blockade and good tolerance in patients with AR+, ER– metastatic BC (Gucalp et al., 2013). Later, a phase II study enrolling patients with locally advanced or metastatic AR+ TNBC, confirmed the antitumor activity and safety of Enzalutamide (Traina et al., 2018). Other studies, including the one by Lu et al. (2019), described the combination of Bicalutamide and Aromatase inhibitor in patients with ER+/AR+ advanced BC. However, in this cohort, it was not reported a synergistic activity, suggesting that more large-sample clinical trials should be managed in this population to better understand how to overcome endocrine resistance (Lu et al., 2019). At present, there are no standard targeted therapies for the treatment of AR+ patients, given that AR is recognized as a protein whose regulation is still under discussion. Targeting the AR signaling could represent one of the challenging approaches to overcome the lack of response to current available therapies, and miRNAs belong to the most appealing candidates to be used for such therapeutic purposes. Here we show that miR-9-5p, which is downregulated in BC cell lines and in primary BC FFPE samples compared to healthy counterparts, downregulates AR, both at the mRNA and at the protein level, and that it is upregulated after androgens stimulation, regardless of the ER status. These findings suggest a possible feedback loop in which AR stimulation induces miR-9-5p in BC cells, which in turn silences AR expression and prevents AR downstream signaling even in presence of AR agonists. To our knowledge, this is the first evidence that demonstrates such an intertwined loop between androgens, miR-9-5p and AR in BC (Supplementary Figure S7). While the pro- or anti-proliferative effect of miR-9-5p in BC is still debated, in our cell line models miR-9-5p elicits an anti-proliferative effect (3 different cell lines and 3 different time points). Interestingly, the anti-tumoral effect of miR-9-5p is independent of the ER status, providing the rationale for the study of this miRNA as a possible therapeutics especially in subtypes of BC such as TNBC, currently with a dismal prognosis. Cell Titer-Glo assay was used to assess the number of viable cells based on the number of metabolically active cells, whereas BrdU incorporation assay was used to obtain quantitative information on cells that are actively replicating their DNA. The different results obtained with the two methods should be interpreted in light of the intrinsic differences between the two assays. We observed a significant decrease of cell proliferation in ER+ cells MCF-7 and T-47D, after DHT exposure, while in ER- cell line MDA-MB-453 although it induced a slight increase of cell metabolic activity, it seemed to decrease the percentage of cells in S phase, overall supporting an antiproliferative effect. It is believed that the androgen/estrogen imbalance can promote tumor progression depending on the predominant hormone. In addition, aromatizable androgens such as androstenedione and testosterone may have both anti-proliferative or pro-proliferative effect depending on several variables, i.e., the activity of estrogen- or androgen- synthesizing enzymes (aromatase and 5α-reductase, respectively), the intracellular ER/AR expression ratio, and the concentration of circulating androgens (Secreto et al., 2019). About the latter, it has been suggested that they could have a role as independent molecules but also as a substrate for estrogen synthesis albeit limited to AR+/ER+ BC (Giovannelli et al., 2018). Interestingly, in the absence of ER-α more than a half of AR binding events had a pattern analogous to that of ER-α in ER+ cells, promoting ER target genes expression and cell growth, thus suggesting a role of AR as a ER-α mimic (Robinson et al., 2011). All together, these findings highlight the need to better understand the androgen/estrogen network, in order to clarify the different behaviors observed in BC subtypes and patients, especially in relation to the presence or absence of ER, as we showed in our data. Since miR-9-5p over-expression decreases BC cell proliferation, our findings rise the very provocative question whether AR antagonists might actually weaken the anti-proliferative effects of miR-9-5p, by preventing androgen-induced up-regulation of miR-9-5p in BC cells. Certainly further studies should be conducted to better understand this point and clarify how miR-9-5p levels and modulation could affect anti-AR treatments in AR+ BC. In support to a possible correlation between miR-9-5p and AR, we further reported an inverse correlation between miR-9-5p and AR expression in a set of paired FFPE tumor and adjacent non-tumor BC patients. Obviously, the small size of our patient cohort represents a limitation of this study, together with the fact that we did not interrogate the effects of miR-9-5p in AR regulation in an *in vivo* model. Future studies will cover these limitations but the fact that an inverse correlation between miR-9-5p and AR expression in primary samples was achieved already in such a small number of patients, should be interpreted as encouraging and superior to any animal model data. In summary, we identified miR-9-5p as a tumor suppressor gene in BC, regardless of ER status, capable of down-regulating AR in BC cells and to inhibit AR downstream signaling even in presence of androgen agonists. We finally report that miR-9-5p is induced by AR agonists, supporting the existence of an androgen/miR-9-5p/AR feedback circuitry that should be accounted for when exploiting AR antagonists as therapeutics for BC patients.

## Data Availability Statement

The raw data supporting the conclusions of this article will be made available by the authors, without undue reservation.

## Ethics Statement

The studies involving human participants were reviewed and approved by Romagna Ethics Committee (C.E.R.O.M.) and IRST IRCCS Medical Scientific Committee (CMS). The patients/participants provided their written informed consent to participate in this study.

## Author Contributions

MF: conceptualization, supervision. EB and FF: data curation, writing–original draft. FF: formal analysis. EB, IV, TR, MP, and FFb: investigation. RM: patients enrollement. MT and FL: immunoistochemistry and slide evaluation. EB: methodology. EB and MF: project administration. FF, IV, FFb, SH, WC, and MF: writing–review and editing. All authors have read and agreed to the published version of the manuscript.

## Conflict of Interest

The authors declare that the research was conducted in the absence of any commercial or financial relationships that could be construed as a potential conflict of interest.

## Abbreviations

AR: Androgen receptor
BC: Breast cancer
EMT: Epithelial-mesenchymal transition
FFPE: Formalin-fixed paraffin-embedded
miRNAsmicroRNAs
DHT: Dihydrotestosterone
DHEA: Dehydroepiandrosterone.
