# Supplementary material for: miR-9-5p as a Regulator of the Androgen Receptor Pathway in Breast Cancer Cell Lines
Source: Front Cell Dev Biol. 2020 Nov 12;8:579160. doi: 10.3389/fcell.2020.579160 (PMC7689370; doi:10.3389/fcell.2020.579160)
Supplement: Supplementary file 1 [file Data_Sheet_1.pdf]

SUPPLEMENTARY MATERIAL

1. Supplementary Tables and Figures

1.1 Tables

SAMPLE	AGE AT DIAGNOSIS	HISTOLOGY	T	N (positive/asported)	M	GRADE	Er (%)	PgR (%)	Her2 amp	Ki67 (%)
BC#1	57	TNBC	2	0 (1)	0	3	0	0	N	55
BC#2	75	LumA	1b	0 (2)	0	2	100	25	N	10
BC#3	43	TNBC	1c	1a (1/35)	0	3	0	0	N	60
BC#5	59	LumA	1b	0(1)	X	3	100	100	N	15
BC#6	43	TNBC	1c	0 (1)	X	3	0	0	N	80
BC#7	53	LumA	2	1a (1/15)	X	2	90	100	N	25
BC#8	57	LumA	1b	0 (1)	X	1	100	80	N	5
BC#9	59	LumA	1c	0 (2)	0	2	100	0	N	15
BC#10	54	LumA	1c	0 (1)	X	2	100	100	N	10
BC#11	40	TNBC	1c	1a (1/19)	0	3	0	0	N	85
BC#12	32	TNBC	1c	0 (N/A)	N/A	3	0	0	N	45

Table S1. Histological and clinical information about BC patients used in this study. According to the TNM Staging System as recommended by The American Joint Committee on Cancer (AJCC) and the International Union Against Cancer (UICC). T1b: 0.5 to 1.0 cm; T1c: 1.0 to 2.0 cm; T2: 2 to 5 cm. N0: no cancer cells in any nearby nodes, pN1a: cancer cells have spread into 1 to 3 lymph nodes and at least one is larger than 2mm; between brackets: (number of nodes analyzed/number of nodes asported). MX: metastasis cannot be measured; M0: cancer has not spread to other parts of the body. N/A: means such information is not available; Er: estrogen receptor; PgR: progesterone receptor; Her2 amp: Her2 gene amplification.

1.2 Figures

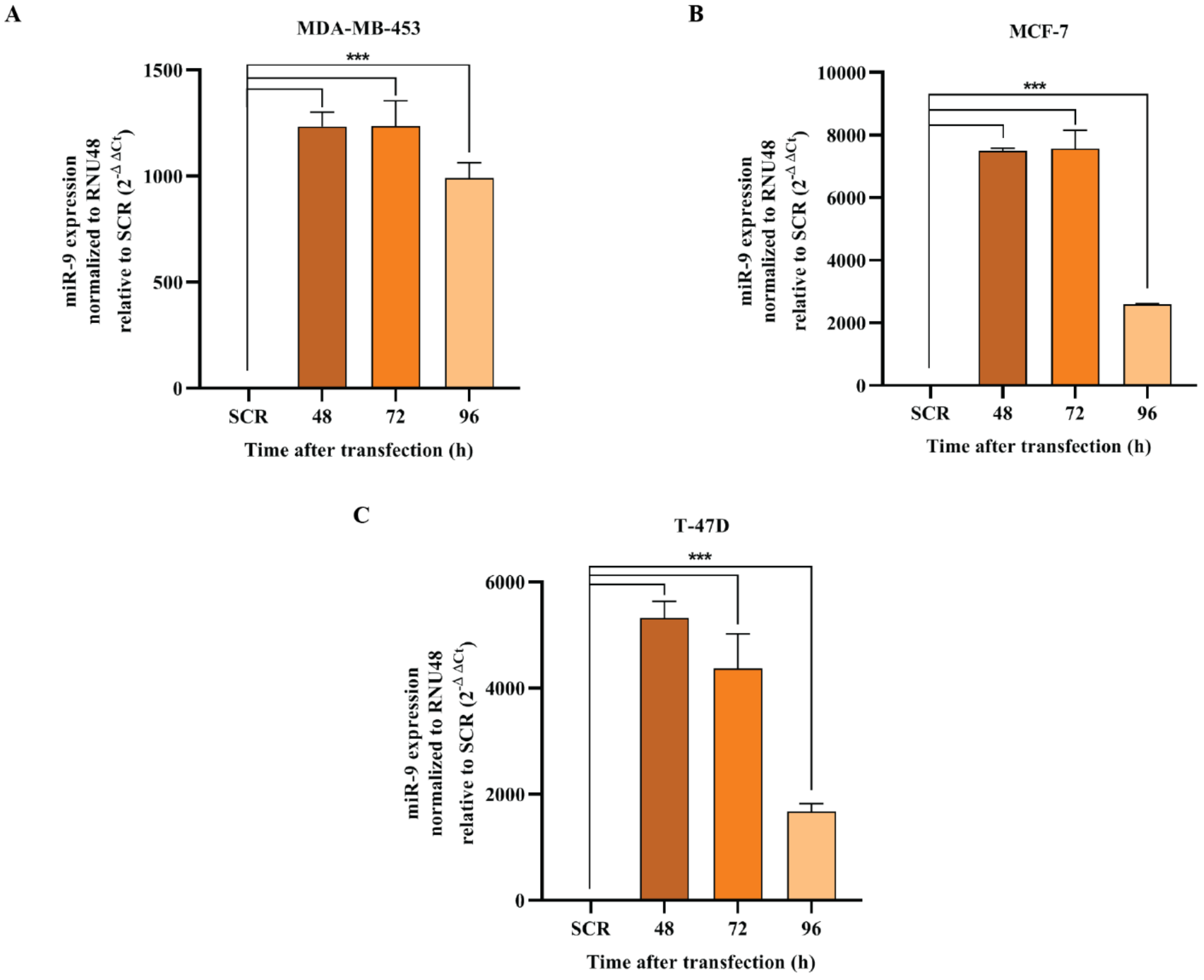

Figure S1. MiR-9-5p (miR-9) levels 48-72-96 h after transfection of MDA-MB-453 (**A**), MCF-7 (**B**), and T-47D (**C**) cells, compared to scrambled (SCR). All data represent means ± SD. *** $p < 0.001$. One-way ANOVA.

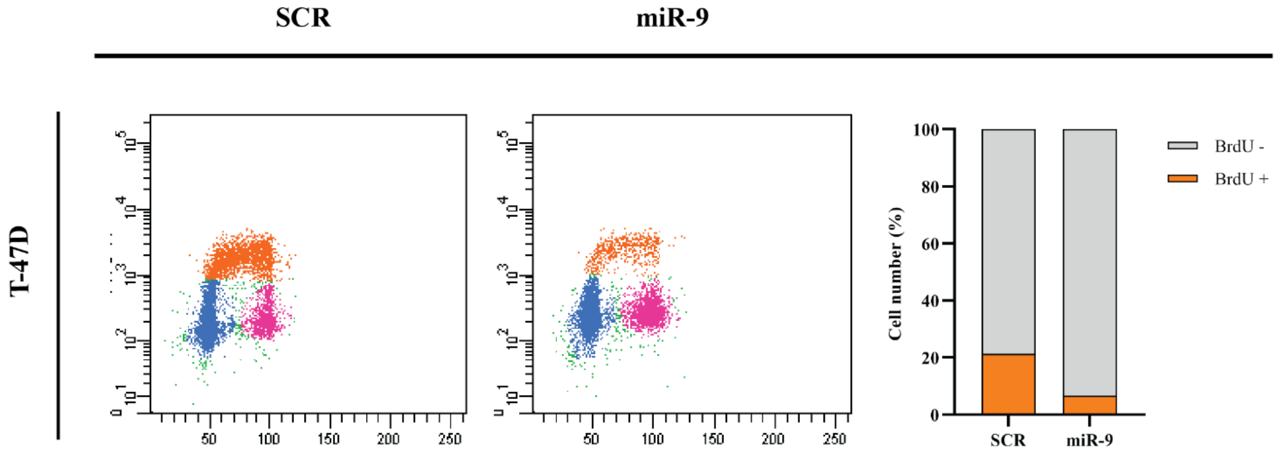

Figure S2. Cytofluorimetric evaluation of the effects of miR-9-5p (miR-9) on DNA replication at 96h post transfection. Cytofluorimetric dot plots of proliferating T-47D BC cell line, following BrdU incorporation, and anti-BrdU antibody incubation. The percentage of cells in S phase, which results in BrdU positive cells (BrdU+) was presented in the histograms on the right.

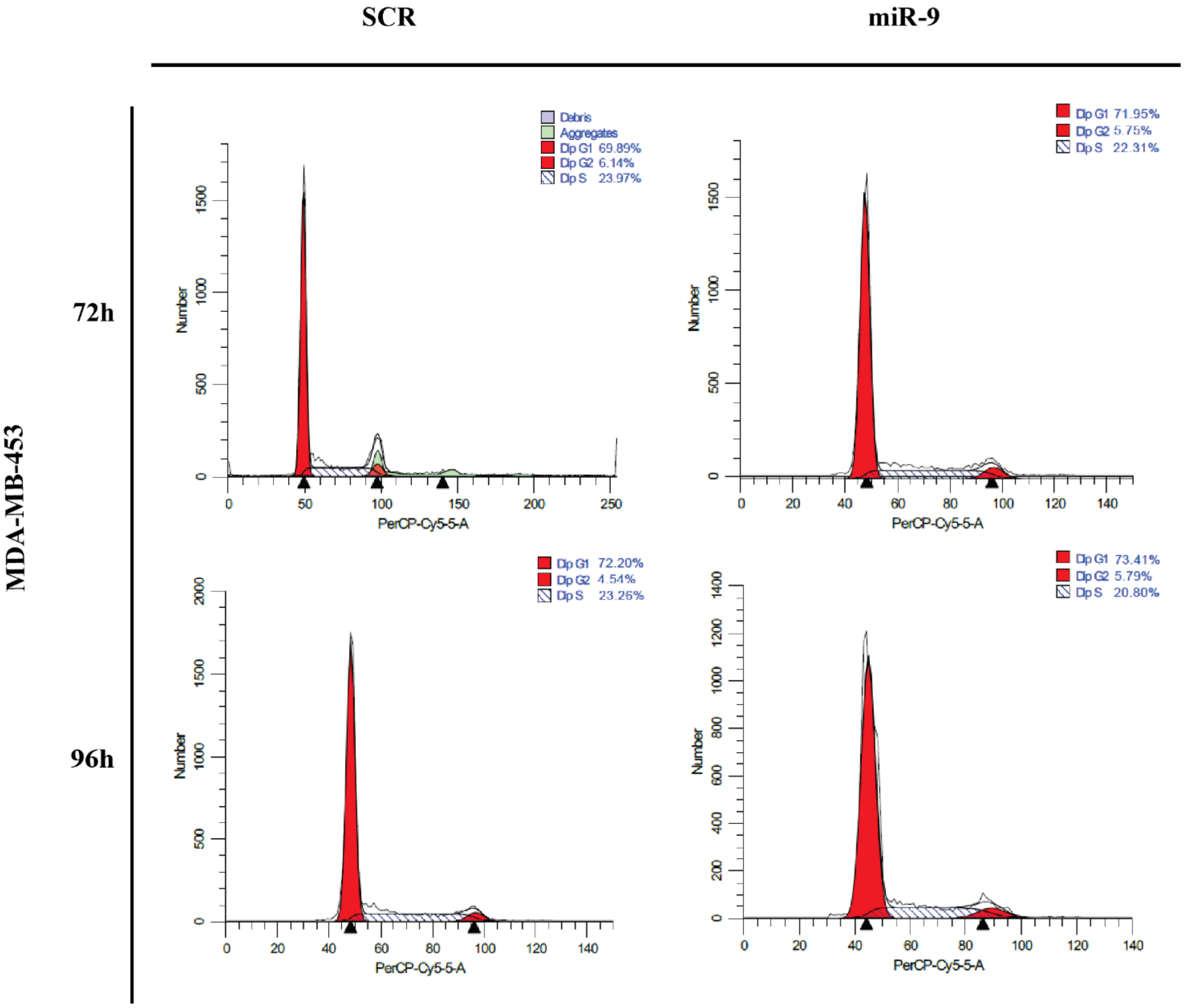

Figure S3. Effect of miR-9-5p (miR-9) compared to negative control (SCR) on cell cycle in MDA-MB-453 breast cancer cell line. Cytofluorimetric analysis of hypotonic propidium iodide-stained cells, with percentage of cell in each cell cycle phases.

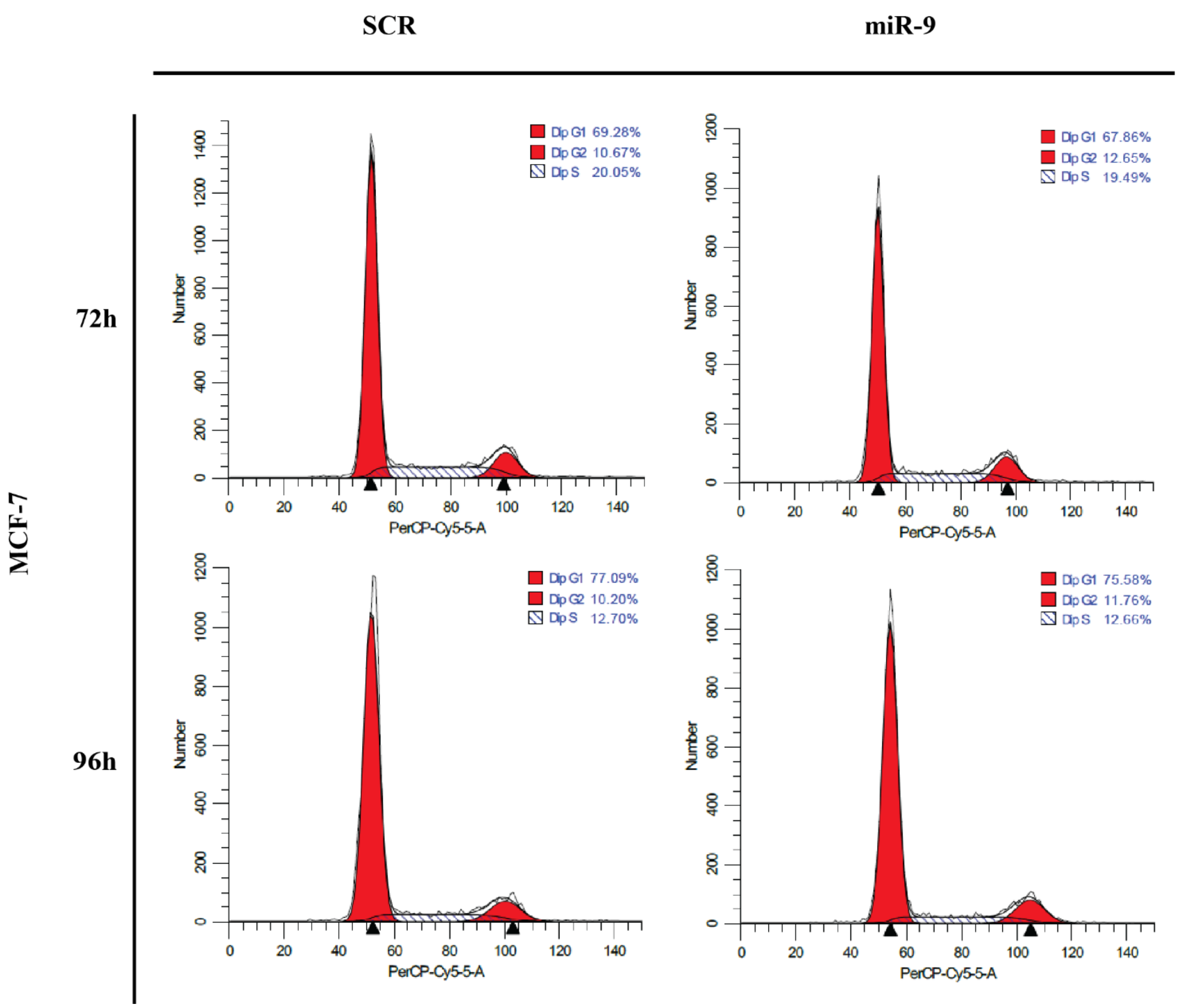

Figure S4. Effect of miR-9-5p (miR-9) compared to negative control (SCR) on cell cycle in MCF-7 breast cancer cell line. Cytofluorimetric analysis of hypotonic propidium iodide-stained cells, with percentage of cell in each cell cycle phases.

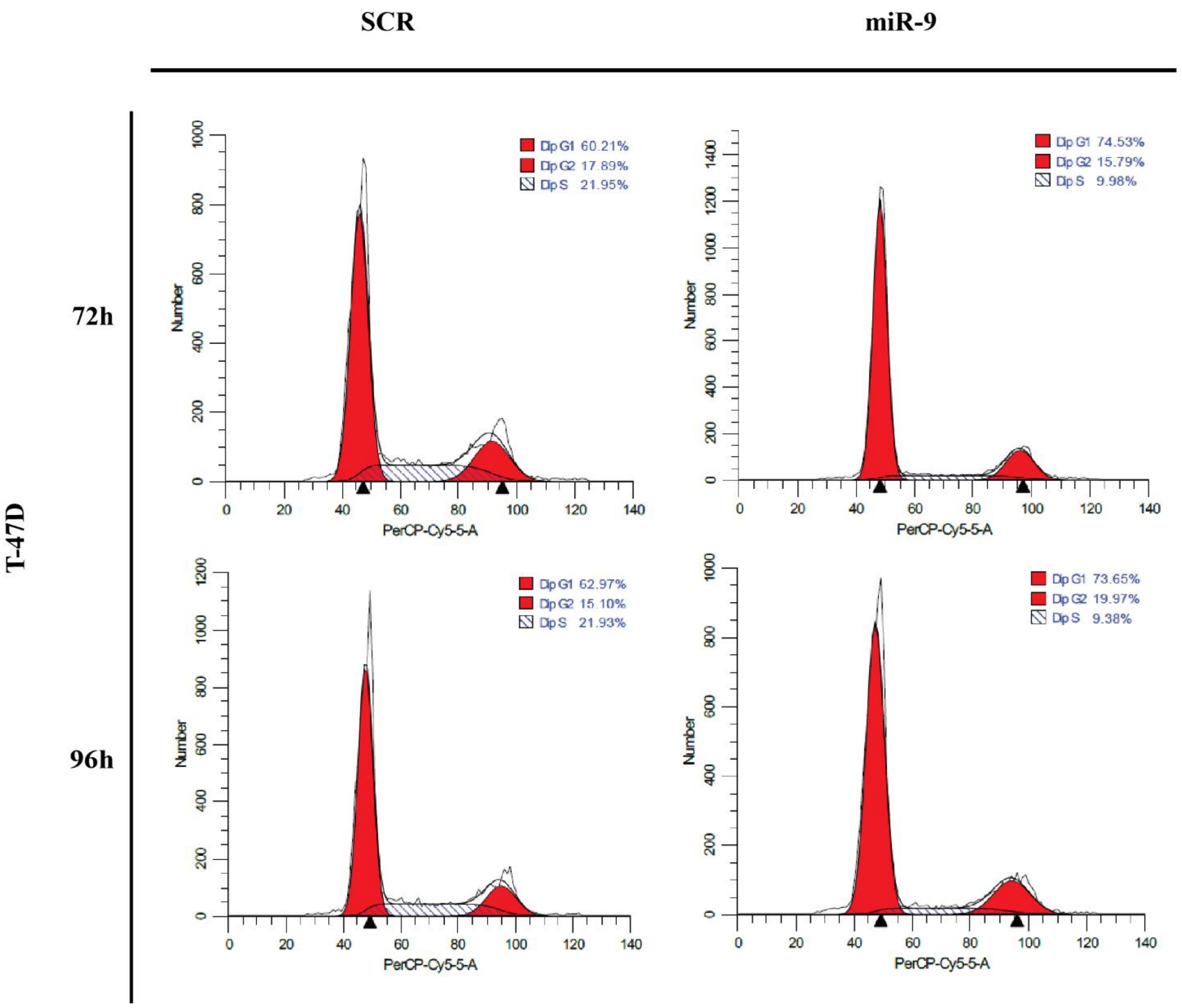

Figure S5. Effect of miR-9-5p (miR-9) compared to negative control (SCR) on cell cycle in T-47D breast cancer cell line. Cytofluorimetric analysis of hypotonic propidium iodide-stained cells, with percentage of cell in each cell cycle phases.

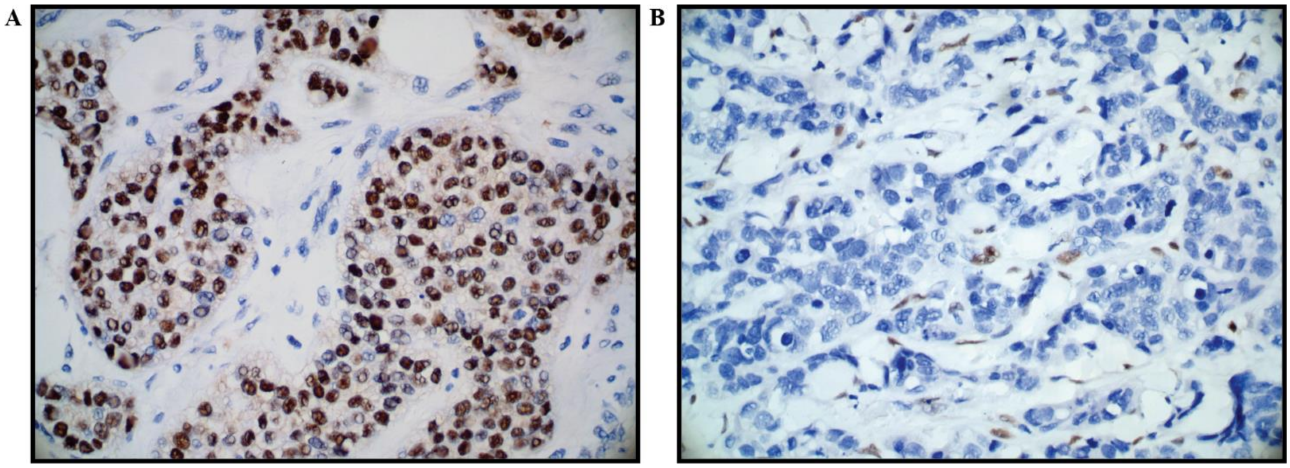

Figure S6. Representative image of FFPE BC samples breast showing positivity (**A**) and negativity (**B**) for AR expression. All 40× magnification.

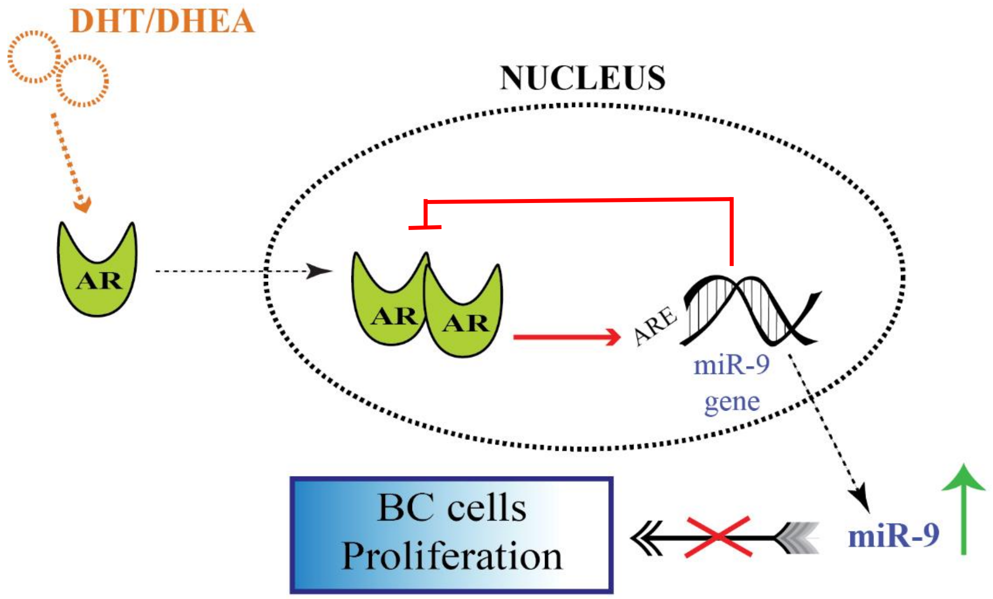

Figure S7. Schematic representation of AR-miR-9-5p interaction in BC.

AR agonists (DHT/DHEA) upregulate miR-9 which, in turn, inhibits AR expression and BC cell proliferation.
